# 沙利度胺联合环磷酰胺和地塞米松治疗症状性华氏巨球蛋白血症的前瞻性临床研究

**DOI:** 10.3760/cma.j.issn.0253-2727.2023.08.012

**Published:** 2023-08

**Authors:** 燕姗 黄, 文婕 熊, 颖 于, 禹廷 阎, 婷玉 王, 瑞 吕, 薇 刘, 刚 安, 耀中 赵, 德慧 邹, 录贵 邱, 树华 易

**Affiliations:** 1 中国医学科学院血液病医院(中国医学科学院血液学研究所)，实验血液学国家重点实验室，国家血液系统疾病临床医学研究中心，细胞生态海河实验室，天津 300020 State Key Laboratory of Experimental Hematology, National Clinical Research Center for Blood Diseases, Haihe Laboratory of Cell Ecosystem, Institute of Hematology & Blood Diseases Hospital, Chinese Academy of Medical Sciences & Peking Union Medical College, Tianjin 300020, China; 2 天津医学健康研究院，天津 301600 Tianjin Institutes of Health Science, Tianjin 301600, China

华氏巨球蛋白血症（WM）是一种异质性很强的疾病，对于有症状或治疗指征的WM患者，国际WM工作组仍首先推荐患者进入设计严谨的临床试验。目前治疗WM的有效药物包括烷化剂苯丁酸氮芥、环磷酰胺、核苷酸类似物氟达拉滨、抗CD20单克隆抗体利妥昔单抗、蛋白酶体抑制剂硼替佐米、布鲁顿酪氨酸激酶抑制剂（BTKi）伊布替尼等[1]–[2]。对于我国WM患者，能规范应用含利妥昔单抗或硼替佐米方案治疗的比例并不高。免疫调节剂沙利度胺成功应用于治疗包括WM在内的多种肿瘤，并显示出良好疗效[3]–[7]，而沙利度胺价格便宜，易于被我国患者接受，故我们在2015年设计本研究探讨沙利度胺联合环磷酰胺和地塞米松治疗症状性WM的疗效。

## 病例与方法

1. 病例：本研究为单中心、前瞻性、单臂临床研究，研究注册号为NCT02844309/ChiCTR-OIC-16008402。纳入2015年1月至2018年4月在中国医学科学院血液病医院确诊的初治/复发症状性WM患者，因经济原因等不能接受含利妥昔单抗或硼替佐米为主方案治疗。入组标准：①年龄≥18岁；②符合WM的诊断标准，且具有治疗指征。排除标准：①1年内曾确诊或治疗过B细胞非霍奇金淋巴瘤以外的恶性肿瘤（包括活动性中枢神经系统淋巴瘤）；②已有临床证据表明发生大细胞淋巴瘤转化；③伴有其他严重疾病，会对该研究造成影响（如未控制的糖尿病、胃溃疡及其他严重的心肺疾病等），判断决定权归属于研究者；④已知的感染人类免疫缺陷病毒（HIV）病史；⑤患者在30 d内曾接受过较大的手术（不包括淋巴结活检）；⑥妊娠期或者哺乳期女性，未采取避孕措施的育龄期妇女；⑦对所用药物过敏。研究方案符合赫尔辛基宣言，且获得本单位伦理委员会批准（BDH-WM2015/02）。入组患者自愿加入临床试验并签署知情同意书。

2. 治疗方案：TCD方案具体用药：沙利度胺起始剂量50 mg/晚，1周后加至100 mg/晚，持续口服；环磷酰胺300 mg/m^2^，口服，第1、8、15天；地塞米松20 mg，口服，第1、2、8、9、15、16天。每（28±3）d为1个疗程，该方案为口服方案，达到微小反应（MR）及以上疗效的患者以TP方案维持，具体用量：沙利度胺100 mg/晚，持续口服；泼尼松0.5 mg/kg 隔日1次口服。

3. 疗效评价：本研究的主要目的为评价TCD方案对症状性WM患者的总体有效率（ORR）；次要终点包括评价无进展生存（PFS）时间及总生存（OS）时间。疗效评价主要参考第六届国际WM工作组的推荐的疗效评价标准[9]。ORR为治疗12个月或<12个月终止用药时的疗效评估结果，最佳疗效为治疗及随访期间全部治疗周期内任一评估中的最优疗效。OS时间指确诊WM至任何原因导致死亡或末次随访的时间，失访患者以末次随访状态为截止数据。PFS时间指淋巴浆细胞淋巴瘤患者治疗至疾病进展、复发、死亡的时间。

4. 安全性评价：根据美国国家癌症研究所制定的常见不良事件评价标准（NCI CTCAE）4.0版对不良事件（AE）进行分级。

5. 随访：采用电话联系的方式随访。随访时间截至2022年5月22日。随访内容包括患者距随访日期最近一次血常规、免疫球蛋白、免疫固定电泳、淋巴结超声及腹部超声等。生存患者的中位随访时间为73.6个月。

6. 统计学处理：采用SPSS 26.0软件进行统计分析。计量资料符合正态分布的以均数±标准差表示，不符合正态分布的以中位数（范围）进行描述。

## 结果

1. 患者的临床特征：11例患者纳入本研究。男8例，女3例，中位年龄为63（40～74）岁，入组时疾病状态：初治患者9例，复发患者2例。ISSWM评分：低危1例，中危3例，高危者7例。其中7例患者进行MYD88L265P及CXCR4基因检测，均为MYD88L265P阳性CXCR4阴性（表1）。

**表1:** 11例采用沙利度胺联合环磷酰胺和地塞米松治疗的症状性华氏巨球蛋白血症患者的临床特征

例号	年龄(岁)	性别	HGB (g/L)	PLT (×10^9^/L)	IgM (g/L)	β_2_微球蛋白(g/L)	M蛋白(g/L)	乳酸脱氢酶(U/L)	ISSWM分组	MYD88突变	CXCR4突变
1	63	男	67	126	118.0	8.08	38.67	122	高危组	阳性	阴性
2	40	男	95	320	48.7	7.96	36.42	112	高危组	/	/
3	58	男	60	62	53.6	/	/	112	中危组	阴性	阴性
4	71	女	96	111	120	3.45	52.00	112	高危组	阴性	阴性
5	42	女	88	149	56.6	6.43	28.82	185	中危组	阳性	阳性
6	56	男	73	99	22.0	6.31	/	133	高危组	/	/
7	63	女	89	323	50.5	2.64	38.17	145	低危组	阳性	阳性
8	60	男	66	164	67.2	3.22	43.62	142	中危组	阳性	阳性
9	70	男	76	128	46.7	3.84	26.72	143	高危组	阳性	阳性
10	72	男	52	165	29.8	/	/	170	高危组	阴性	阴性
11	74	男	95	190	34.8	5.21	18.16	168	高危组	阴性	阴性

注 /：未检测

2. 疗效和生存分析：中位治疗12（3～36）个月，4例患者治疗超过2年。11例患者中，有9例患者达到MR及以上疗效，其中包括2例患者血清M蛋白下降超过75％，6例患者血清M蛋白下降超过50％，ORR为81.9％。中位起效时间为5（2～12）个月，11例患者经TCD方案治疗后血红蛋白、血小板及免疫球蛋白得到不同程度改善，其中10例患者血红蛋白升高至100 g/L及以上，8例患者IgM下降超过50％，1例患者IgM下降大于25％。中位随访73.6个月，11例患者中共6例出现疾病进展；6例死亡，其中4例死于本病，2例因二线治疗方案不良反应死亡。中位PFS时间46.4个月，中位OS时间81.8个月（图1）。

**图1:**
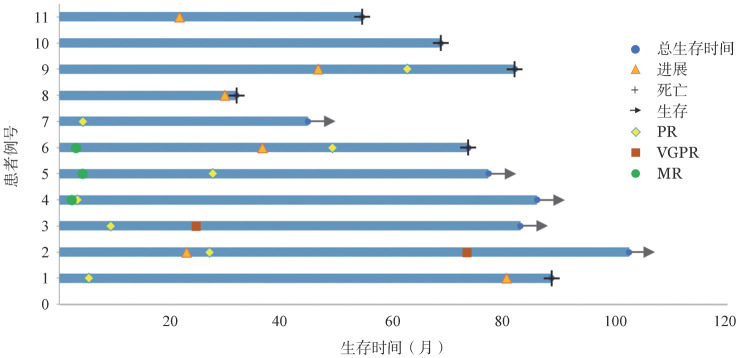
沙利度胺联合环磷酰胺和地塞米松方案治疗11例症状性华氏巨球蛋白血症患者生存及疗效变化 PR：部分缓解；VGPG：非常好的部分缓解；MR：微小反应

3. 安全性：11例接受该方案治疗的患者中以乏力、头晕等贫血症状起病患者8例，出血、水肿、球蛋白升高起病各1例，无以周围神经损害起病入院患者。使用沙利度胺治疗期间均未出现血红蛋白和血小板计数下降等血液学不良反应，主要的不良反应为周围神经损害，其中1例患者因Ⅲ/Ⅳ级周围神经病变停药；Ⅰ/Ⅱ级非血液学不良反应包括周围神经病变2例、便秘1例、下肢水肿2例，均经停药或对症治疗后好转。

## 讨论

WM是一种罕见的惰性淋巴瘤，综合国内外治疗指南[1],[8]，对于有症状的WM患者一线治疗首先建议入组临床试验。沙利度胺是一种免疫调节剂，其抗肿瘤机制目前仍不明确，可能的作用机制包括抑制血管生成、调节参与肿瘤细胞和骨髓基质相互作用的黏附分子，以及调节影响肿瘤存活的细胞因子等[7],[9]。相关研究表明，沙利度胺在WM中具有杀伤肿瘤的作用，且联合治疗有效率优于单药治疗。沙利度胺的抗肿瘤作用最早是在多发性骨髓瘤（MM）中被发现[5]，在MM的治疗中不仅具有高反应率且不良反应少[10]，因此不少研究者将其应用于WM的治疗中。国外的一项研究，纳入20例有治疗指征的WM患者，使用单药沙利度胺以起始剂量200 mg/d口服，最大耐受量为600 mg治疗，治疗结束5例患者达部分缓解（PR），ORR为25％[11]。在另一项研究中，使用沙利度胺联合利妥昔单抗治疗，纳入28例有症状的WM患者，起始予沙利度胺200 mg/d，联合8次利妥昔单抗，治疗52周；治疗结束64％的患者获得PR以上疗效，ORR为72％[12]。相比沙利度胺单药治疗，联合治疗的有效率得到很大提升。

采用TCD方案患者的生存较采用以利妥昔单抗为基础的治疗方案者未见明显差异。本研究中，中位治疗12个月，ORR为81.9％，中位随访73.6个月，中位PFS时间46.4个月，中位OS时间81.8个月。Dimopoulos等[13]的研究纳入72例有症状的WM患者，采用利妥昔单抗联合环磷酰胺及地塞米松（RCD方案）治疗，中位治疗4.1个月，ORR为83％，中位随访23.4个月，2年PFS率为 67％，2年OS率为81％；另一项研究纳入23例有症状的WM患者接受利妥昔单抗联合硼替佐米及地塞米松（BRD方案）治疗，中位治疗7个疗程，ORR为96％[14]。两项研究有效率优于TCD方案，但均出现了治疗后血液系统损害。靶向药物治疗时代，BTKi在WM治疗中广泛应用，一项伊布替尼单药治疗初治WM患者的研究纳入30例初治WM患者，接受伊布替尼单药治疗48个月，ORR为100％，MR率为83％，18个月PFS率为92％[15]。另一项研究者发起的研究纳入63例既往治疗过的WM患者，使用伊布替尼单药治疗26个月，ORR为90％，MR率为77.7％，2年PFS率及OS率分别为69.1％和95.2％[16]。两项研究均获得了很好疗效，但同样出现了较为严重的不良事件。

TCD方案可以为有治疗指征的WM患者提供一种治疗选择。与联合化疗及BTKi单药治疗相比，本研究中不良反应主要与沙利度胺的剂量限制毒性相关，主要为外周神经炎表现，且相关的研究证明增加沙利度胺剂量与患者的总体有效率无关[11]，因此在本研究中沙利度胺起始剂量50 mg/晚，最大量为100 mg/晚并延长患者服药时间，以达到最佳疗效。本研究中出现的不良反应以周围神经损害为主，且多数患者可耐受，对症治疗或停药后均有好转。总体而言，TCD治疗方案的不良反应较小，目前尚未出现不可控的不良事件。

尽管TCD方案安全有效，但仍有不足之处。该方案最大不足为目前尚无研究证明服用沙利度胺达到缓解后患者可以安全停药，因此TCD是长期口服治疗方案。另外TCD方案起效慢，中位起效时间为5（2～12）个月，深度缓解率低（18.18％）。本研究纳入因经济等原因不能接受含利妥昔单抗、硼替佐米或BTKi治疗方案的患者，因此病例数有限，结论需进一步增大样本量后进行验证。对于复发难治的患者，目前国内外指南均推荐BTKi治疗[2]，但对于经济条件不佳者，TCD方案具有重要应用价值。

综上，TCD方案对于WM患者具有较好的疗效及安全性，且TCD为口服治疗方案，价格便宜，患者依从性强，是经济条件欠佳或一般状况差不适合住院化疗患者的重要选择。该方案易于在基层推广使用，期待未来在更大样本中验证。
